# Time-Course of the Effects of QSYQ in Promoting Heart Function in Ameroid Constrictor-Induced Myocardial Ischemia Pigs

**DOI:** 10.1155/2014/571076

**Published:** 2014-04-10

**Authors:** Qi Qiu, Yang Lin, Cheng Xiao, Chun Li, Yong Wang, Kexu Yang, Wei Suo, Yu Li, Wenjing Chuo, Yongxiang Wei, Wei Wang

**Affiliations:** ^1^Capital Medical University Beijing An Zhen Hospital, Beijing 100029, China; ^2^China-Japan Friendship Hospital, Beijing 100029, China; ^3^Beijing University of Chinese Medicine, Beijing 100029, China

## Abstract

We aim to investigate the therapeutic effects of QSYQ on a pig myocardial ischemia (MI) model and to determine its mechanism of action. The MI model was induced by Ameroid constriction of the left anterior descending coronary (LAD) in Ba-Ma miniature pigs. Four groups were created: model group, digoxin group, QSYQ group, and sham-operated group. Heart function, Ang II, CGMP, TXB_2_, BNP, and cTnT were evaluated before (3 weeks after operation: 0 weeks) and at 2, 4, and 8 weeks after drug administration. After 8 weeks of administration, the pigs were sacrificed for cardiac injury measurements. Pigs with MI showed obvious histological changes, including BNP, cTnT, Ang II, CGRP, TXB_2_, and ET, deregulated heart function, and increased levels of apoptotic cells in myocardial tissue. Treatment with QSYQ improved cardiac remodeling by counteracting those events. The administration of QSYQ was accompanied by a restoration of heart function and of the levels of Ang II, CGRP, TXB_2_, ET BNP, and cTnT. In addition, QSYQ attenuated administration, reduced the apoptosis, and decreased the level of TNF-**α** and active caspase-3. In conclusion, administration of QSYQ could attenuate Ameroid constrictor induced myocardial ischemia, and TNF-**α** and active caspase-3 seemed to be the critical potential target of QSYQ.

## 1. Introduction

Cardiac dysfunction caused by myocardial ischemia is the difficulty in clinical treatment. Clinical studies suggest that diuretics and vasodilators are able to improve the clinical symptoms of patients with cardiac insufficiency. ACEI (angiotensin-converting enzyme inhibitor), *β*-receptor blockers, and aldosterone receptor antagonists can improve the prognosis. However, positive inotropic agents and vasodilators can improve the clinical symptoms at an early stage, but long-term use can lead to increased mortality, and some drugs may also decrease the survival rate [1]. Angiotensin-converting enzyme inhibitors (ACEI) and *β*-receptor blockers, though they improve myocardial function in the long term, inhibit myocardial remodeling and increase left ventricular ejection fraction (LVEF) and do not have beneficial effects in the treatment of early-stage hemodynamic disorders [2, 3]. In recent years, the study of traditional Chinese medicine (TCM) prevention and treatment of heart failure has led to a large number of basic and clinical research results based on complex components of many molecular targets [4, 5].

Apoptosis is suggested to be a key event in ischemia-reperfusion injury, resulting in LV dysfunction, remodeling, and heart failure [6, 7]. One study found that, in patients with end-stage heart failure, the incidence of myocardial apoptosis was 0.08–0.25%, while the normal myocardium apoptosis rate was 0.001-0.002% [8]. Moderate and sustained levels of myocardial apoptosis may lead to heart failure [9]. Therefore, drugs that inhibit cardiomyocyte apoptosis in patients with residual cardiac function may be protective and may delay and inhibit the progression of heart failure. Ginseng,* Carthamus tinctorius*, and other traditional Chinese medicines and their active ingredients can inhibit cardiomyocyte apoptosis after ischemia [10, 11]. Qin et al. have reported that inhibition of cell apoptosis significantly improves heart function in myocardial infarction rabbits [12]. In particular, TNF-*α*/caspase-3-mediated apoptosis is considered the main apoptotic pathway in heart failure (HF) [13]. Therefore, in vivo detection of apoptosis may not only prove clinically useful in diagnosis and prognosis but also indicate that the TNF-*α*/caspase-3 pathway is potential therapeutic target [14].

Qishenyiqi (QSYQ) is a traditional Chinese medicine that has long been used for the routine treatment of coronary heart disease (CHD) and chronic heart failure (CHF) in China [15]. It consists of six Chinese herbs (Radix Astragali Mongolica,* Salvia miltiorrhiza* bunge, FlosLonicerae, Poria, Radix Aconiti Lateralis Preparata, and Radix Glycyrrhizae) and is widely produced in China in accordance with the Chinese Pharmacopoeia standard of quality control [15]. Our previous study found that QSYQ ameliorates myocardial hypertrophy and remodeling by inhibiting the expression of AngII in LAD rats [16]. However, little is known about the exact targets of QSYQ in its effects on myocardial remodeling. The purpose of the present study is to investigate whether QSYQ treats HF by improving left ventricular remodeling associated with apoptosis.

## 2. Materials and Methods

### 2.1. Materials

This study used the following materials: Ameroid constrictor, 2.75 mm diameter (Research Instrument SW, USA); anerdian (Shanghai Likang Disinfectant High-Tech limited, batch no. 20050808); ketamine hydrochloride injection (Jiangsu Hengrui Medicine Company Limited, lot no. KH050302); diazepam injection (Tianjin Jin Hui Amino Acid Company Limited, lot no. 0506131); benzylpenicillin sodium for injection (North China Pharmaceutical Company Limited batch no. D0406207); Ultravist 370 iopromide injection (Ernst Schering (Guangzhou) Pharmaceutical Co., Ltd., lot no. 43617); meglumine diatrizoate injection (X-ray contrast agent) (Shanghai Asahi Donghai Ching Pharmaceutical, batch no. 041003); apoptosis detection kit (In Situ Cell Death Detection kit, POD Roche, Germany, Cat. no. 11 684-910); disodium hydrogen phosphate (Beijing Eddie Fine Chemicals Limited, lot no. 990,329); sodium dihydrogen phosphate (Beijing Hongxing Chemical Plant, lot no. 870,509); formaldehyde (Beijing Chemical Reagent Company, batch no. 03030); Lys-C (Roche, Germany); modified trypsin (Roche, Germany); BCA protein Quantification kit, 5× SDS-protein sample preparation fluid, concentrated gel buffer solution, separation gel buffer solution, 30% bis-acrylamide/acrylamide, cellulose nitrate film, and Kodak films (Beijing Pulilai Genetic Technologies Limited); the phosphatase inhibitor PMSF and protease inhibitors (Nanjing KGI Biotechnology Developments Limited); EasySee Western Marker (20 kDa–90 kDa) (Beijing-Gold Biotechnology Company); rabbit polyclonal TNF-*α* antibody (Abcam, USA, cat. no. Ab6671); rabbit polyclonal TNF receptor I antibody (Abcam, USA, cat. no. Ab19139); rabbit polyclonal active caspase-3 antibody (Abcam, USA, cat. no. Ab13847); rabbit polyclonal caspase-8 antibody (Abcam, USA, cat. no. Ab15552); mouse monoclonal [6C5] GAPDH-antibody (Abcam, USA, cat. no. Ab8245); and ECL plus (GE, USA; cat. no. 2134).

### 2.2. Animal Handling Procedure

Forty male Ba-Ma miniature pigs (20 ± 2 kg) were purchased from the Institute of Experimental Animals in the Fuwai Hospital Experimental Animal Center. The pigs were housed under standard laboratory conditions, fed three times a day, and given tap water ad libitum. Experimental procedures were reviewed and approved by the Animal Care and Use Committee and the Ethics Committee in the Fuwai Hospital before the animal experiments were carried out.

### 2.3. Surgical Protocol

**Figure 1:**
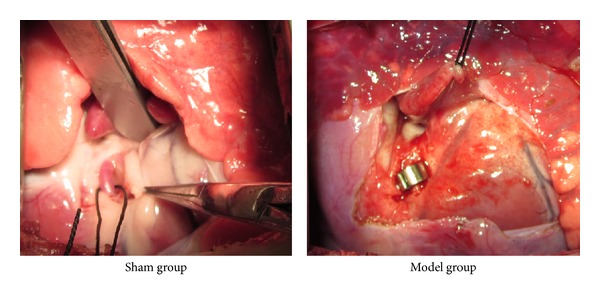
Model of Ameroid constrictor-induced MI caused cardiac dysfunction in pigs.

After 1 week of dietary modification, all animals underwent the surgical procedure. The day before surgery, all animals received aspirin (325 mg orally) and were fasted for 12 h. Prior to surgical procedure, all animals received prophylactic antibiotics and buprenorphine (0.03 mg/kg, intramuscular) for pain control. All survival procedures were conducted in a sterile fashion. For all surgical procedures, animals were given general anesthesia after sedation with Telazol (4 mg/kg, intramuscular), followed by endotracheal intubation and ventilation with a volume-cycled ventilator (North American Dragger). General anesthesia was maintained with a gas mixture of oxygen at 1.5–2 L/min and 3% isoflurane. The animal's vital signs were recorded during surgery and throughout postoperative recovery. Femoral access via a percutaneous stick was achieved for arterial access, blood draws, and blood pressure monitoring. An Ameroid constrictor (internal diameter 2.75 mm) was placed on the proximal left circumflex coronary artery (LCx) via small left thoracotomy through the fourth intercostal space (Figure 1). Sham-operated animals (*n* = 6) received no Ameroid ring, but other operations were identical to the model group (Figure 1).

Thirty minutes prior to the end of each procedure, a dose of buprenorphine (0.03 mg/kg, intramuscular) was administered. After each procedure, the dosage of aspirin was continued for five days, while a fentanyl patch (4 *μ*g/kg) was applied for 72 h for pain control.

### 2.4. Preparation and Dose Calculation of Concentrated QSYQ

The QSYQ used in the present study was manufactured by Beijing University of Chinese Medicine (Beijing, China) using the six constituent Chinese herbs at a composition of 460 g Radix Astragali Mongolica, 230 g* Salvia miltiorrhiza* bunge, 160 g Flos Lonicerae, 160 g* Scrophularia*, 140 g Radix Aconiti Lateralis Preparata, and 90 g Radix Glycyrrhizae [14, 17]. Briefly, following extraction with 95% ethanol, the residue of Radix Astragali Mongolica was mixed with* Salvia miltiorrhiza* bunge, Flos Lonicerae,* Scrophularia*, and Radix Glycyrrhizae, followed by extraction with hot water (twice, 2 h each). The water extract was then concentrated to form a paste, and ethanol was added for 24 h. We then collected the filtrate to form the final product. In the present study, we used the dose of 0.33 g/kg, which was based on the clinical application dose of 20 g per day per 60 kg body weight.

### 2.5. Experimental Groups and Drug Treatment

All the model animals underwent echocardiography at the end of the third week after operation and were randomly assigned to 3 experimental groups (*n* = 8): control group, a group treated with digoxin (0.0123 mg/kg body weight per day, Shanghai Pharmaceutical Group Co., Ltd., the Hsin Yi Pharmaceutical Factory, batch no. 100505), and a group treated with QSYQ (0.33 g/kg body weight per day). The medication started on the day after evaluation (the first day of the fourth week after operation) and continued to the end of the twelfth week after operation (lasting 8 weeks).

### 2.6. Echocardiography

Transthoracic echocardiography of all the animals was performed using an Agilent SONOS 5500 before medication and at 2, 4, and 8 weeks after medication. Three-dimensional biplane Simpson's method [18] was used to measure the LV end-diastolic volume (LVEDV) and LV end-systolic volume (LVESV). LVEDV was assumed to be the largest volume, and LVESV was chosen as the smallest volume. LV stroke volume (LVSV) was calculated as the difference between diastolic and systolic volume, and LVEF was calculated as ratio of LVSV to LVEDV.

### 2.7. Measurement of CGRP, AngII, TXB_2_, ET, BNP, and cTnT by Radioimmunoassay (RIA)

The plasma (1 mL) was homogenized in saline containing enzyme inhibitor (10 *μ*L of 0.3 M EDTA-Na, 10 *μ*L of 0.34 M 8-hydroxyquinoline, and 5 *μ*L of 0.32 M dimercaptopropanol) on ice. The homogenate was centrifuged at 8000 ×g for 10 min. The supernatant was used for determination of calcitonin gene-related peptide (CGRP), angiotensin II (AngII), thromboxane B_2_ (TXB_2_), endothelin (ET), brain natriuretic peptide (BNP), and troponin T (cTnT), using a RIA kit (Beijing Kangyuan Ruide Biotechnology Co., Ltd., Beijing, China) following the manufacturer's instructions.

### 2.8. Histology

The ventricles were fixed in 4% paraformaldehyde, and paraffin-embedded hearts were sectioned at 200 mm intervals from base to apex, and serial sections of 4 mm were cut and placed on polylysine-coated glass slides. Tissue sections were deparaffinized and stained with Masson's trichrome reagent.

### 2.9. TUNEL Method

For in situ detection of DNA fragmentation in paraffin-embedded tissue sections, the TUNEL method was performed using the In Situ Cell Death Detection kit, POD (Roche Molecular Biochemicals, Indianapolis, IN) following the manufacturer's instructions. Briefly, sequential 4 *μ*m tissue sections were adhered to silane-coated slides and allowed to dry at RT. Subsequently, sections were deparaffinized and rehydrated. Proteins were digested by incubating tissue sections in 20 *μ*g/mL proteinase K (Worthington Co., Lakewood, USA) for 15 min at room temperature (RT). Endogenous peroxidase was inactivated with 2% H_2_O_2_ in distilled water (dH_2_O) for 5 min at RT. The labeling mixture containing biotinylated dUTP in TdT enzyme buffer was added to sections and incubated at 37°C in a humidified chamber for 1 h. After stopping the enzymatic reaction, sections were rinsed with PBS for 15 min, covered with anti-digoxigenin peroxidase conjugate, and incubated for 30 min at RT in a humidified chamber. Then, sections were incubated in TBS with 0.05% diaminobenzidine (DAB) plus 3% H_2_O_2_ until color development. Sections were rinsed in PBS, dehydrated, and mounted. In control sections in which the enzyme TdT was omitted from the reaction solution, no stained nuclei were observed.

### 2.10. Transmission Electron Microscope Test

From each group the cardiac tissues were minced into small pieces (≤1 mm^3^) and fixed in 2.5% glutaraldehyde in 0.1 mol/L sodium cacodylate buffer (pH 7.3) for 2 hrs. The specimens were rinsed in buffer, postfixed in cacodylate-buffered 2% OsO4, stained en bloc in uranyl acetate, dehydrated gradient in ethanol, and embedded in epoxy resin. Finally, 50–70 nm super thin slices were prepared, stained with uranyl acetate and lead citrate, and examined under an electron microscope (JEOL-1230, Japanese Electronics Company, Japan).

### 2.11. Heart Preparation and Protein Extraction

The heart was excised and incubated in ice-cold PBS to wash out blood at the end of 8 weeks after medication (*n* = 6). Each left ventricle was then carefully dissected to remove all the necrotic/scarred zones to keep only the viable myocardium in the marginal zone of the infarct region in model animals. The left ventricular myocardium below the ligation in sham animals was also dissected. The samples were then immediately frozen in liquid nitrogen and stored at −80°C.

Frozen tissue from 6 animals of each group was ground to a powder with liquid nitrogen using a mortar and pestle. Tissue extraction medium (40 mM Tris-HCl pH 7.4, 7 M urea, 2 M Thiourea, 1% w/v DTT, and 1 mM EDTA) (1 : 10) and protease inhibitor cocktail (1 : 50) were then added to the powder, and the mixture was ultrasonicated for 30 s. Ten microliters of RNase and 5 *μ*L DNase were added, and the reaction was incubated on ice for 20 min. The samples were centrifuged at 12,000 ×g at 4°C for 20 min to remove the insoluble material. After centrifugation, the protein concentration of each sample was quantitated using a Bradford assay and used for subsequent analysis.

### 2.12. Western Blot Analysis

Protein samples were prepared as described above and subjected to western blot analysis. The samples (50 *μ*g) were separated by SDS-PAGE (12.5% or 15% gel) at 100 V for 2 h. Gels were then transferred to a NC membrane that had been presoaked for 10 s in transfer buffer (25 mM Tris, pH 7.4, 192 mM glycine, and 20% methanol) at 300 mA for 1.5 h or 2 h. Each NC membrane was blocked for 2 h with 0.5% dried skim milk in TBS-T (20 mM Tris, 500 mM NaCl, and 0.05% v/v Tween 20) at RT, washed three times for 15 min each with TBS-T, and then incubated with a specific primary antibody (anti-TNF-*α*, anti-TNFR1, anti-active caspase-3, anti-caspase 8, and anti-GAPDH, Abcam, USA) in TBS-T with gentle shaking overnight at 4°C. Each membrane was washed three times for 15 min each with TBS-T and then incubated with the secondary antibody (horseradish peroxidase-labeled goat anti-rabbit IgG) in TBS-T with gentle shaking at 37°C for 1 h. Each membrane was rinsed three times for 15 min each with TBS-T, developed with the Super Enhanced Chemiluminescence Detection kit (GE healthcare, USA). All the membranes were exposed in and scanned by a ChemiDoc XRS+ (Bio-Rad). A semiquantitative analysis based on the OD values was performed by Image Lab Software (Bio-Rad), using ANOVA to compare groups.

### 2.13. Statistical Analysis

Statistical analysis was performed with SPSS version 17.0. All data are presented as the mean ± standard deviation (SD). Statistical analysis was carried out on three or more groups using one-way analysis of variance (ANOVA) and Dunnett's test. Values of *P* < 0.05 were considered statistically significant.

## 3. Results

An Ameroid constrictor-induced Ba-Ma pig model of myocardial ischemia was used in this study. Due to differences in the coronary artery diameter in Ameroid ring models, the judgment of the administration time is important. Our previous study found that, 3 weeks after surgery, the left coronary artery of model animals was 100% blocked, which suggests the formation of myocardial ischemic lesions. We choose 4 weeks after operation to administer drugs in this study, with digoxin as the control drug. Macroscopic signs of the animals in each group, cardiac function, and endocrine-related indicators were monitored before administration and at 2, 4, and 8 weeks after administration.

### 3.1. QSYQ Improved Heart Function in Ameroid Induced CHF Pigs

Time-course echocardiography was used to assess the heart function in the four groups to evaluate the role of QSYQ in preventing heart from myocardial ischemia. In comparison with the sham group, myocardial ischemia caused a significant decline in LVEF and an increase in LVSV and LVDV (Table 1), indicating an impairment of heart function. Treatment with QSYQ at 0.33 g/kg and digoxin protected against this impairment; furthermore, the improvement of EF% of QSYQ was significant when compared with digoxin treatment.

### 3.2. Serum Levels of CGRP, TXB_2_, AngII, ET, BNP, and cTnT

**Figure 2:**
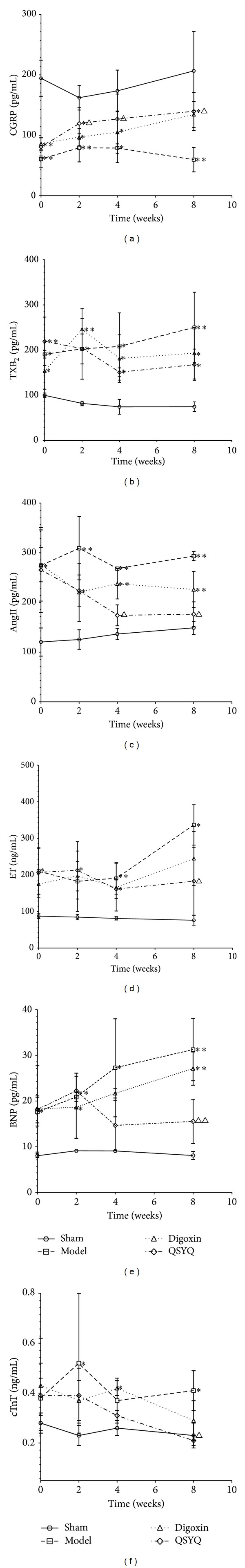
CGRP, TXB_2_, AngII, ET BNP, and cTnT in plasma as measured by RIA (*n* = 6). QSYQ: Qishenyiqi. *P < 0.05 compared with sham group, **P < 0.01 compared with sham group, ^Δ^ *P* < 0.05 compared with model group, and ^ΔΔ^ *P* < 0.01 compared with model group.

The time-course RIA analysis of CGRP, TXB_2_, AngII, ET, BNP, and cTnT showed that myocardial ischemia caused the increase of serum TXB_2_, AngII, cTnT, ET, and BNP (*P* < 0.01 or *P* < 0.05, Figures 2(b), 2(c), 2(d), 2(e), and 2(f)). The increase of AngII, ET, BNP, and cTnT was significantly suppressed by QSYQ treatment (*P* < 0.01 or *P* < 0.05, Figures 2(c), 2(d), 2(e), and 2(f)). Digoxin treatment showed decreased trend but without significant changes. However, the opposite was true for CGRP. CHF pigs treated with QSYQ and digoxin had significantly lower CGRP compared to model pigs (*P* < 0.01 or *P* < 0.05, Figure 2(a)).

### 3.3. QSYQ Prevents Pathological Changes of Cardiac Tissue in MI Pigs

**Figure 3:**
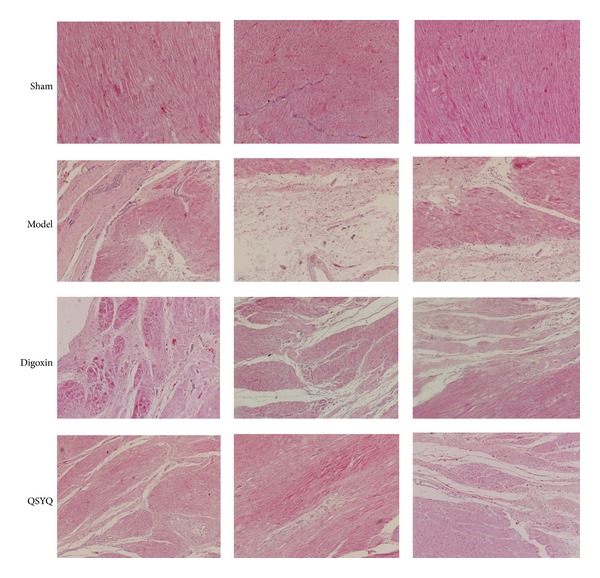
Morphology (×200). HE results in different groups. Sham: sham group. Model: model group. Digoxin: digoxin group. QSYQ: QSYQ group.

**Figure 4:**
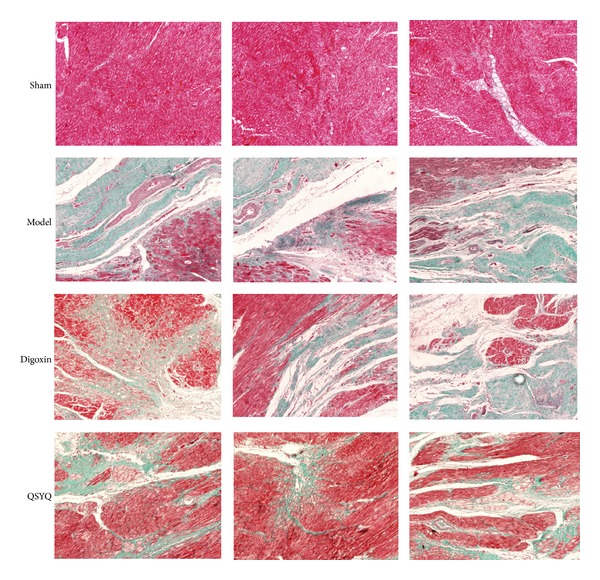
Morphology (×200). Masson results in different groups. Sham: sham group. Model: model group. Digoxin: digoxin group. QSYQ: QSYQ group.

To investigate the roles of QSYQ in protecting CHF, we initially examined the heart morphological changes in the different groups. Eight weeks after drug administration, pathological examination showed that the cardiac myocytes exhibited an irregular shape and arrangement, with myocardial fibrosis (Figure 3). Masson's trichrome staining showed that the fibrotic area was significantly greater in the model group than in the sham group (Figure 4). Moreover, the number of cardiac myocytes was greatly reduced. Notably, QSYQ treatment significantly suppressed these phenotypes in the model animals and decreased the fibrotic areas, while digoxin treatment did not suppress those phenotypes.

### 3.4. QSYQ Treatment Inhibits Apoptosis in CHF Pigs after MI

**Figure 5:**
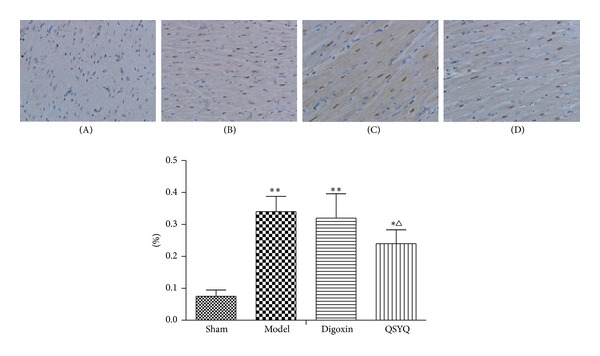
QSYQ inhibits apoptosis in pigs with HF. TUNEL analysis was carried out 8 weeks after drug treatments. (A) TUNEL-positive cells (apoptotic cells) in the sham group; (B) model; (C) digoxin; (D) QSYQ. *P < 0.05 compared with sham group, **P < 0.01 compared with sham group, ^Δ^ *P* < 0.05 compared with model group, and ^ΔΔ^ *P* < 0.01 compared with model group.

Apoptosis is one of the major outcomes of CHF. Our previous findings motivated us to further investigate the impact of QSYQ on myocardial cell apoptosis. By TUNEL assay, we found that increased numbers of apoptotic myocardial cells were present in CHF pigs, whereas QSYQ treatment dramatically decreased the apoptosis rate (Figure 5). Digoxin did not decrease the apoptosis rate significantly when compared with model group.

### 3.5. Transmission Electron Microscopy Results of the Heart Tissue in Different Groups

**Figure 6:**
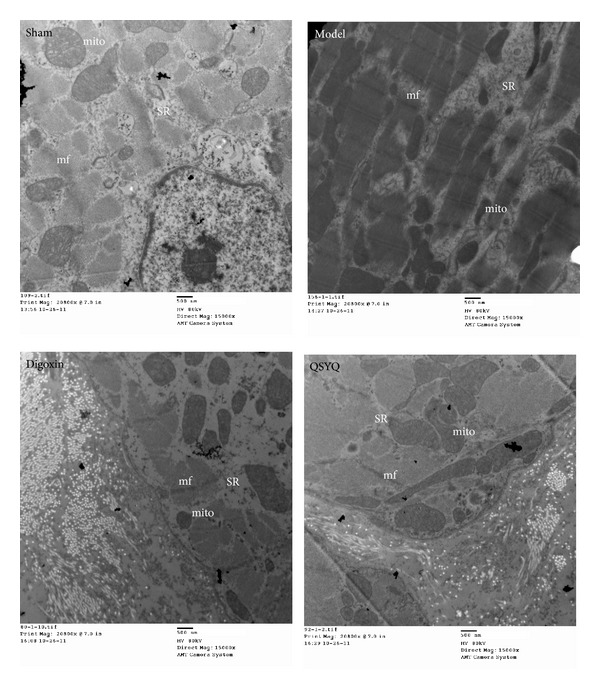
Ultrastructure changes. Sham: sham group. Model: model group. Digoxin: digoxin group. QSYQ: QSYQ group. SR: sarcoplasmic reticulum; mito: mitochondria; and mf: myofibril.

Normal cardiac ultrastructure was shown in sham group. Pyknotic mitochondria and myogenic fragmentation are observed in model and digoxin group. QSYQ treatment prevented mitochondria pyknotic and myogenic fragmentation, but digoxin did not (Figure 6).

### 3.6. QSYQ Treatment Decreases TNF-*α* and Active Caspase-3

**Figure 7:**
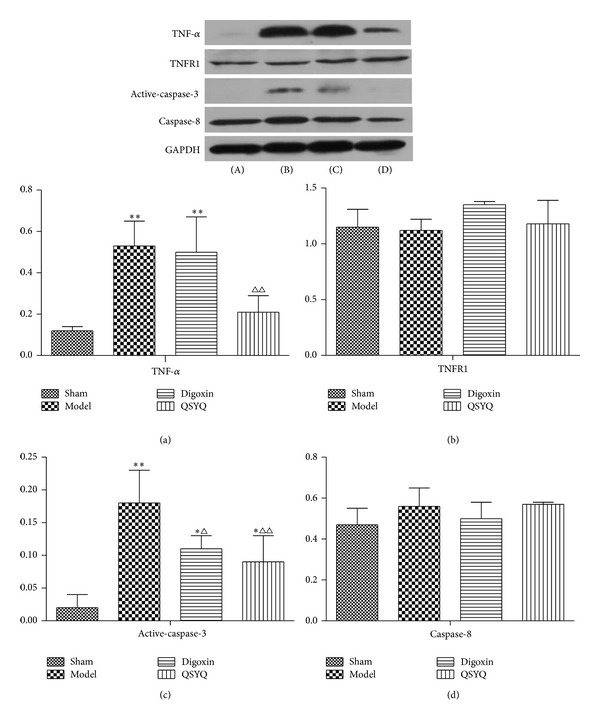
The western blot results of (a) TNF-*α*, (b) TNFR1, (c) active caspase-3, and (d) caspase-8 in the four groups (*n* = 6), (A) sham group, (B) model group, (C) digoxin group, and (D) QSYQ group. *P < 0.05 compared with sham group, **P < 0.01 compared with sham group, ^Δ^ *P* < 0.05 compared with model group, and ^ΔΔ^ *P* < 0.01 compared with model group.

TNF-*α*, TNFR1, active caspase-3, and caspase-8 levels are critical in the prognosis for myocardial apoptosis [19, 20]. Western blots showed that cardiac TNF-*α*, TNFR1, active caspase-3, and caspase-8 (Figure 7) in the Control group increased (*P* < 0.01) compared with the sham-operated group, while treatment with QSYQ for 8 weeks reduced TNF-*α*, active caspase-3, and caspase-8 compared with the Control group (*P* < 0.05), to a level similar to sham group (Figure 7). Digoxin treatment inhibited the caspase-3 activation but did not work on TNF-*α* expression.

## 4. Discussions

Improving heart function is the most important part of treatment of CHF. Digoxin is one of the most widely used drugs which functions by inhibiting the *α*-subunit of cell-membrane Na^+^, K^+^-ATPases, promoting Na^+^-Ca^2+^ exchange, and increasing intracellular Ca^2+^ concentration, which acts on the contractile proteins, resulting in enhanced myocardial contractility [21]. In addition, digoxin can improve CHF patients' baroreceptor sensitivity and inhibit AV nodal conduction. Digoxin increases LVEF and improves hemodynamic parameters in a dose-dependent manner, but it increases the mortality of female patients [22]. Based on the exact cardiac function improvement efficacy, we chose digoxin as a positive control to observe the onset time and degree of heart function improvement of QSYQ.

The present study showed that, as the treatment time increased, QSYQ-treated pigs showed a gradual trend of recovery of heart function and significant improvement in myocardial injury indicators. The main clinical manifestations of cardiac insufficiency after myocardial ischemia are decreases in myocardial contractility, left ventricular pump function, and cardiac output, resulting in inadequate organ perfusion. The end-diastolic volume is a reflection of both structural remodeling and diastolic filling (end-diastolic myocyte fiber length). The end-systolic volume is influenced by both the end-diastolic volume and fiber shortening, but asymmetric contraction may make echocardiographically derived measures of end-systolic volume inaccurate. Although heart rate and fiber shortening both affect ejection fraction, ejection fraction is influenced to a far greater extent by end-diastolic volume because changes in stroke volume tend to be much smaller than changes in end-diastolic volume [23]. BNP, a polypeptide consisting of 32 amino acids, is mainly secreted by the ventricles with the feature of explosive synthesis and short half-life, and shows sensitivity and specificity to ventricular dysfunction. As the ventricular volume and pressure load are proportional and closely related to left ventricular function, BNP has become an important biochemical indicator of the level of cardiac function in CHF patients [24, 25]. cTnT is a specific structural protein, a subunit of troponin complexes in myofibrillar filaments with calcium binding sites, that is primarily involved in the activation and regulation of calcium ions in the process of muscle contraction. Approximately 95% of the cTnT is present in the muscle fiber, and 5% of cTnT is secreted from the myocardial cytoplasm into the blood under physiological conditions. This free cTnT in the blood significantly increases when myocardial cells are injured [26]. The concentration of cTnT in the blood and the degree of myocardial injury are positively correlated with each other and negatively correlated with congestive heart failure [27].

CGRP, AngII, TXB_2_, and ET are vasoactive substances, which can regulate vascular contraction and the myocardial oxygen supply. CGRP is composed of 37 amino acids and is a powerful arteriovenous vascular relaxant. It is more sensitive in the small vessels and is the most powerful and most enduring relaxing factor, as it increases adenylate cyclase (Ac) activity and increases cAMP reserves in vascular endothelial cells or vascular smooth muscle cells [17, 28]. ET is a potent vasoconstrictor of 21 amino acids that is synthesized and secreted by endothelial cells, and its abnormal concentration is a sign of vascular endothelial cell injury. Under physiological conditions, ET concentration is low, and CGRP is in a state of dynamic equilibrium. The two are mutually antagonistic in pathological states [29]. Angiotensin II is a major peptide in the renin-angiotensin system. Its biological effects include blood vessel contraction, fibrinolysis inhibition, and tissue fibrosis promotion. It plays an important role in the progression of coronary heart disease. Myocardial ischemia and hypoxia can increase the activity of the RAS system and increase circulating AngII, which may be an important indicator of the presence of coronary heart disease and blood stasis [30]. TXB_2_ is the stable metabolite of TXA_2_ and has been used to represent the levels of TXA_2_ in clinical situations. Plasma TXB_2_ is significantly increased in heart failure patients, and benazepril can reduce it [31]. TXA_2_ is a metabolite of arachidonic acid and is synthesized and released by platelets. It has powerful vasoconstrictor activity by promoting platelet aggregation and thrombus formation. TXA_2_ decreases cAMP in platelets and vascular smooth muscle cells by inhibiting AC or as a Ca^2+^ carrier that directly contributes to Ca^2+^ influx and dense pipeline system Ca^2+^ release, which promote platelet aggregation, local vasoconstriction, and endothelial damage [32]. The present study showed that QSYQ can regulate the expression of these substances and protect the myocardium from hypoxic injury.

Another major finding in this study was that QSYQ can significantly decrease the apoptosis rate by downregulating both TNF-*α* and active caspase-3, but no changes of TNFR or caspase-8 were detected in the myocardial infarct border zone. TNF-*α* is an important inflammatory factor that mediates the development of inflammation. In the myocardial ischemia process, TNF-*α* can inhibit myocardial contraction, promote myocardial hypertrophy, and induce apoptosis of cardiac cells. The TNF-*α*/TNFR1 extrinsic pathway of apoptosis is an important sensor signal [17]. Binding of Fas ligand to cell-surface Fas causes a conformational change to the intracellular death domain (DD) of Fas, causing it to bind to a Fas-associated death domain (Fas associated via death domain, FADD) adapter protein, to form DISC protein complexes [33]. Activated procaspase-8 and procaspase-10 are recruited to DISC [34], which activates procaspase-triggered apoptosis. Caspases have numerous important roles in the regulation of apoptosis. Both endogenous and exogenous apoptosis pathways are activated by caspase family members, including caspase-8, caspase-9, and caspase-3, while the antiapoptotic effects of caspase inhibitors have been confirmed. Caspase-3 plays an important role as a substrate of apoptosis and has been used as a sign of irreversible apoptosis. In addition to apoptosis-induced biological effects, caspase-3 can also shear the muscle fibers cells *α*-actin and troponin T [35], and *α*-actin and muscle fiber rupture further reduce contractile function.

## 5. Conclusion

**Figure 8:**
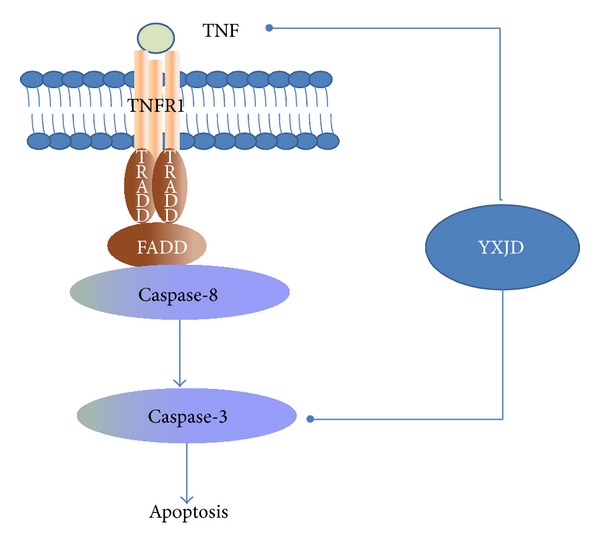
Potential mechanism by which QSYQ attenuates cell death signaling pathways in cardiac dysfunction.

This paper presents a multitarget pharmacological study of the Chinese herbal formula QSYQ. This TCM with multiple chemical components targets multiple proteins, which may produce greater efficacy and fewer side effects than any single constituent. Our results also show that therapeutic QSYQ administration can regulate vasoactive factors to improve myocardial oxygen supply, reduce myocardial injury, improve cardiac function, and inhibit myocardial apoptosis through decreasing the level of TNF-*α* and active caspase-3 (Figure 8).

## Figures and Tables

**Table 1:** The comparison of echocardiography assessment of left ventricular function at each time point in each group ($\overset{-}{x}\pm s$).

Time	Group	*n*	LVSV mL	ΔLVSV mL	LVDV mL	ΔLVDV mL	EF%	ΔEF%
Before administration	Sham	4	6.51 ± 2.40	—	26.70 ± 8.27	—	76.93 ± 4.22	—
	Model	6	20.05 ± 2.08*	—	43.18 ± 4.08	—	51.85 ± 7.50**	—
	Digoxin	6	27.53 ± 12.97**	—	51.73 ± 21.38*	—	47.90 ± 14.07**	—
	QSYQ	6	29.30 ± 7.93**	—	46.77 ± 11.41*	—	36.90 ± 12.70**	—

2 weeks after administration	Sham	4	5.76 ± 1.83	−0.75 ± 0.57	25.20 ± 4.06	−1.50 ± 12.33	77.68 ± 3.33	0.75 ± 0.89
	Model	6	20.00 ± 5.04*	−0.05 ± 2.963	39.40 ± 12.15	−3.78 ± 8.07	46.05 ± 7.55**	−5.80 ± 0.05
	Digoxin	6	20.67 ± 9.67*	−6.86 ± 3.30^∗▵▵^	46.00 ± 15.30*	−5.73 ± 6.08	56.78 ± 15.04**	8.88 ± 0.97^∗∗▵▵^
	QSYQ	6	25.53 ± 5.78*	−3.77 ± 2.15	47.97 ± 7.82	1.20 ± 3.59	47.13 ± 3.99**	10.23 ± 8.71^▵▵▴^

4 weeks after administration	Sham group	4	6.77 ± 2.85	0.26 ± 0.45	30.63 ± 7.12	3.93 ± 15.39	79.05 ± 5.61	10.23 ± 9.78
	Model group	6	22.85 ± 5.31**	2.80 ± 3.23	50.08 ± 11.76*	6.90 ± 7.68	50.34 ± 7.44**	−1.51 ± 0.06
	Digoxin group	6	20.83 ± 9.22*	−6.70 ± 3.75^∗▵▵^	44.95 ± 12.43	−6.78 ± 8.95^∗∗▵▵^	55.23 ± 15.95**	7.33 ± 1.88^∗∗▵▵^
	QSYQ	6	20.67 ± 12.65*	−8.63 ± 4.72^▵^	44.97 ± 21.84	−1.80 ± 10.43^∗∗▵▵^	55.80 ± 8.23**	18.90 ± 4.47^∗▵▵▴▴^

8 weeks after administration	Sham group	4	7.10 ± 3.18	0.59 ± 0.78	28.98 ± 6.98	2.28 ± 15.25	74.88 ± 6.84	18.90 ± 12.03
	Model group	6	21.88 ± 7.65**	1.83 ± 5.57	46.88 ± 13.52*	3.70 ± 9.44	50.71 ± 8.97**	−1.14 ± 1.47
	Digoxin group	6	18.03 ± 6.20*	−9.50 ± 6.77^∗∗▵▵^	42.75 ± 7.67	−8.98 ± 13.71	53.98 ± 14.74**	6.08 ± 0.67^∗∗▵▵^
	QSYQ	6	17.81 ± 8.49	−11.49 ± 0.56^∗∗▵▵^	30.07 ± 10.09	−16.70 ± 1.32^∗∗▵▵^	55.87 ± 5.02*	18.97 ± 7.68^∗∗▵▵▴▴^

Note: *P < 0.05 and **P < 0.01 compared with sham group; ^▵^ *P* < 0.05 and ^▵▵^ *P* < 0.01 compared with model control group; and  ^▴^ *P* < 0.05 and  ^▴▴^ *P* < 0.01 compared with digoxin group.

ΔLVSV = LVSV_*t*_  −  LVSV _before administration_, ΔLVDV = LVDV_*t*_  −  LVDV _before administration_, and ΔEF% = EF%_*t*_  −  EF% _before administration_.
